# Editorial Comment to Varicocelectomy Restores Spermatogenesis in Fontan‐Associated Varicocele: A Two‐Case Series

**DOI:** 10.1002/iju5.70230

**Published:** 2026-07-14

**Authors:** Teppei Takeshima

**Affiliations:** ^1^ Department of Urology, Center for Reproductive Medicine Yokohama City University Medical Center Kanagawa Japan

1

Advances in congenital heart surgery have transformed the prognosis of patients with complex congenital heart disease. As increasing numbers of patients with Fontan circulation now survive into adulthood, issues beyond survival—including fertility and reproductive health—have become increasingly relevant. Nevertheless, male reproductive function remains a relatively neglected aspect of long‐term Fontan care.

In this issue, Tominaga et al. [1] describe two infertile men with Fontan circulation who developed bilateral varicocele and underwent microsurgical varicocelectomy. Although limited to two cases, the report is noteworthy because it introduces a potentially overlooked cause of male infertility in this growing patient population and raises important questions regarding its management.

For andrologists, the most remarkable finding is the reappearance of ejaculated sperm after varicocelectomy in a patient who had progressed to azoospermia. In daily clinical practice, azoospermia is often regarded as evidence of severe or irreversible testicular dysfunction. The present report reminds us that this assumption may not always be correct. At least in selected patients with Fontan circulation, impaired spermatogenesis may be partly attributable to a potentially reversible hemodynamic abnormality.

The pathophysiological rationale is also compelling. Chronic systemic venous hypertension is a hallmark of Fontan physiology and is responsible for a variety of well‐recognized complications, including Fontan‐associated liver disease and peripheral venous abnormalities. Previous studies have demonstrated lower‐extremity venous reflux in a substantial proportion of adult Fontan patients. However, the possible impact of chronic venous congestion on the pampiniform plexus and testicular function has received little attention. In this regard, the concept of Fontan‐associated varicocele proposed by the authors provides a useful framework linking Fontan physiology with male infertility.

An additional strength of this report is that it highlights an important principle in male reproductive medicine: potentially correctable causes of infertility should be carefully evaluated before proceeding directly to sperm retrieval procedures or assisted reproductive technologies. While techniques such as testicular sperm extraction have expanded reproductive options for men with severe male factor infertility, identifying and treating reversible pathology remain an equally important goal. The recovery of ejaculated sperm in one of the present cases illustrates the potential value of this approach.

At the same time, the report raises several unresolved questions. Because elevated venous pressure persists even after surgery, recurrence patterns may differ from those observed in the general varicocele population. Indeed, recurrence was documented in both patients. The true prevalence of Fontan‐associated varicocele, its natural history, and the durability of surgical outcomes remain unknown. Future studies should also explore the role of detailed scrotal ultrasonography, sperm DNA fragmentation testing, endocrine evaluation, and optimal perioperative anticoagulation strategies.

Despite its limitations as a two‐case series, this report broadens our understanding of the reproductive consequences of Fontan circulation and introduces a clinically relevant concept that deserves further investigation. As more Fontan survivors enter their reproductive years, closer collaboration between congenital cardiologists, reproductive urologists, and fertility specialists will become increasingly important. Most importantly, this report reminds clinicians that even severe semen abnormalities, including azoospermia, may not always be irreversible in men with Fontan circulation.

## Conflicts of Interest

The author declares no conflicts of interest.

## Data Availability

Data sharing not applicable to this article as no datasets were generated or analyzed during the current study.
